# Modeling and Characterization of a Kinetic Energy Harvesting Device Based on Galfenol

**DOI:** 10.3390/ma12193199

**Published:** 2019-09-29

**Authors:** Carmine Stefano Clemente, Daniele Davino

**Affiliations:** 1 Department of Energy, Systems, Territory and Construction Engineering, University of Pisa, 56122 Pisa, Italy; 2 Department of Engineering, University of Sannio, 82100 Benevento, Italy; davino@unisannio.it

**Keywords:** energy harvesting, magnetostrictive materials, Galfenol

## Abstract

The proposal of Energy Harvesting (EH) techniques and devices has experienced a significant growth over the last years, because of the spread of low power electronic devices. Small ambient energy quantities can be recovered through EH and exploited to power Wireless Sensor Networks (WSN) used, for example, for the Structural Health Monitoring (SHM) of bridges or viaducts. For this purpose, research on EH devices based on magnetostrictive materials has significantly grown in the last years. However, these devices comprise different parts, such as a mechanical system, magnetic circuit and electrical connections, which are coupled together. Then, a method able to reproduce the performance may be a handy tool. This paper presents a nonlinear equivalent circuit of a harvester, based on multiple rods of Galfenol, which can be solved with standard circuit simulator. The circuital parameters are identified with measurements both on one rod and on the whole device. The validation of the circuit and the analysis of the power conversion performance of the device have been conducted with different working conditions (force profile, typology of permanent magnets, resistive electrical load).

## 1. Introduction

The goal of Energy Harvesting (EH) techniques is to scavenge small environmental energy quantities and convert them into electrical one in order to supply low-power consumption electronics [1,2,3]. Environmental energy is often present in form of mechanical vibrations, thermal gradients, pressure gradients and solar radiations. It is generally accepted that this energy go lost because of its relative expensiveness to be exploited on a large scale. On the other hand, ambient energy could represent a good candidate as a source for remote and harsh applications of Wireless Sensors Network (WSN).

Indeed, in recent decades, the technological development of Information and Communications Technology (ICT) has led to the massive diffusion of portable devices, such as mobile phones, music players, PDAs, tablets, wrist watches, smart watches and, lately, sensors devices used to monitor and control sensitive quantities (temperature, pressure, displacement, etc.), leading to the Internet of Things (IoT) devices. Among several applications, Wireless Sensors Network (WSN) has an important one in the Structural Health Monitoring (SHM) to monitor existing aged structures, such as bridges, viaducts or railways [4,5,6,7,8,9,10,11]. These may be placed in rural areas, where often the electrical network is not present. Then, these devices are supplied from batteries that should be regularly substituted or recharged from the power grid. This is a limitation in the application of WSN to continuous SHM because the maintenance cost of the sensors is considered high and this results in a detriment of users safety.

The advantages of EH is the possibility to minimize the frequency maintenance of batteries (charging or replacing) or even to eliminate this cost for the whole device’s lifetime. This would be very beneficial to power several nodes in a network, used to monitor physical or environmental conditions of buildings [12,13,14]. In particular, in structure such as bridges, viaducts or railways, the energy is present in form of mechanical vibrations due to the ongoing traffic and, therefore, it can be extract and converted with suitable techniques [15,16,17] (Kinetic Energy Harvesting, KEH).

In order to power supply WSNs, a promising EH techniques could be the use of smart materials [18]. In particular, for KEH purposes, the most used are piezoelectric materials, which couple mechanical properties with electrical ones and magnetostrictive materials, which relate mechanical properties with magnetic ones. The latter are of a particular interest, because device based on magnetostrictives show better mechanical characteristics and higher energy density than piezoelectrics and other EH techniques. Moreover, they show a larger magneto-mechanical coupling, no depolarization and creep phenomena and longer life-time with respect to piezoelectrics [19]. The kinetic energy conversion [20,21] takes advantage of the inverse magnetostrictive, or Villari, effect. It consists of the variation of the magnetic induction inside the magnetostrictive material as a result of a variation in the applied mechanical stress. Then, according to Faraday’s law, a coil placed around the “active” material shows a voltage at the terminals, because of the variation of the linkage magnetic flux. This effect can be used to supply in-situ a single node of a WSN for SHM, by coupling it to a rechargable battery or to a super-capacitor. For example, by considering a viaduct, the harvester could be installed, with some technical efforts, below the thermal expansions joints or into the structural bearings. In the first case, the ongoing traffic vehicles would be the source of vibration, with a behavior similar to a non-periodic train of impulses. The second typology would results in a low frequency behavior (related to the structure resonance, typically much lower than 100 Hz) but almost continuous in time.

Among the magnetostrictive materials, Galfenol is favored to be used with respect to Terfenol-D for EH purposes. In fact, the former has better magnetic and mechanical characteristics than Terfenol-D, which is fragile and shows lower saturation induction [22,23]. Moreover, Galfenol exhibits narrow hysteresis cycle and low coercive field [24,25,26], that is, low energy conversion losses.

The aim of this paper is to present, characterize and model a KEH system based on multiple stress-annealed (SA) Galfenol rods. In particular, the device belongs to the force-driven category and it is conceived to be located under the road paving of a bridge or viaduct [16].

In this paper, in Section 2 an experimental setup devoted to characterize the magnetostrictive materials is described and the general behaviors of SA Galfenol are highlighted. In Section 3 an analytical nonlinear fully-coupled model is obtained and used to develop an equivalent electrical circuit of a single rod KEH concept device where the whole mechanical, magnetic and electric quantities are suitably related. Finally, this three port equivalent circuit has been used on a more engineered KEH system based on three rods of SA Galfenol. Simulations and comparison with experimental tests in different working conditions have been performed and discussed in Section 4.

## 2. Experimental Setup and Static Magneto-Mechanical Characteristics of Galfenol

The active core of the KEH device studied in this work is Galfenol SA [24]. It is an Iron-Gallium alloy (Fe_81.6_Ga_18.4_) and it is textured polycrystalline, grown with free-standing zone melt (FSZM) technique by TdVib LLC. Table 1 lists some of Galfenol’s magnetic and mechanical properties. It shows a good combination of magnetic, mechanical and magnetostrictive properties that common smart materials do not. In particular, it exhibits magnetostriction values of about 200–250 ppm but has better magnetic characteristics than Terfenol-D, which makes it particularly suitable for sensing and harvesting use. Gallium concentration strongly affects both the magnetic and magnetostrictive behavior, as reported in Reference [24]. Galfenol’s ability to be used both in tension and compression, robustness, mechanical workability and high Curie temperature (∼ 600 °C) is attracting interest for the alloy’s use in harsh environments. Applications actively being investigated include transducers for down-hole use, fuel injectors, sensing and EH devices. Since the beginning of 2000, the use of stress annealing on Terfenol-D has been studied with the aim of impart a stress “frozen” into material. Stress annealing is a post-manufacturing process and it constitutes a company’s sensitive information. However, the process mainly consists in the applications of high magnetic field and temperature to the material, for a certain time interval. After that, a built-in stress remains in the treated sampled which generates an uniaxial anisotropy that is desirable because it can greatly simplify the design of devices by obviating the need of a pre-stress mechanism [27,28,29]. It is worth to highlight that the Galfenol samples used in this work and the annealing process have been made and conducted, respectively, by TdVib LLC.

**Figure 1:**
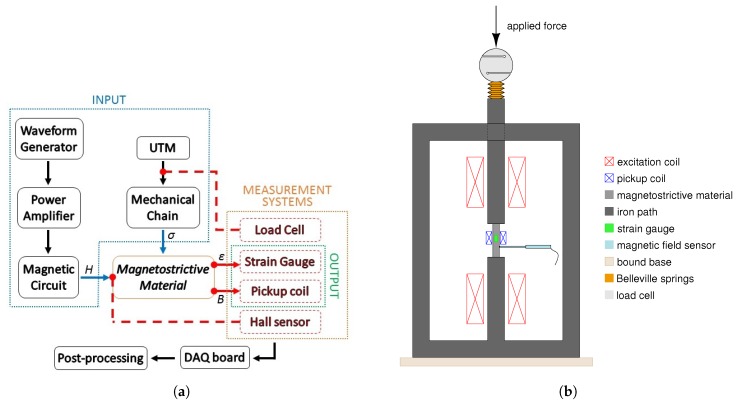
Illustration of the setup used for the characterization of magnetostrictive material. (**a**) Blocks diagram. (**b**) 2-D sketch.

In order to model Galfenol SA, its experimental characterization is strictly necessary. Then, in the following it is described the experimental setup developed and realized for this purpose and the main characteristics and properties of Galfenol are measured and discussed. It is worth to note that, in an overall vision, magnetostrictive materials could be seen then as a 2 input–2 output system. In particular, by applying to the material one mechanical (*stress*, σ) and magnetic (*field*, *H*) input it responses with the corresponding mechanical (*strain*, ε) and magnetic (*magnetization*, *M*) output. A schematic blocks diagram of the experimental setup is shown in Figure 1a. In the central part of Figure 1a is the magnetostrictive material where the blue arrows represent the two input, while the red ones the output. The red dots and text box represent the measurements points and the measurement systems respectively. A 2-D sketch of the setup used for magnetostrictive materials characterization is represented in Figure 1b.

More in details, the magnetic field is provided by two excitation coils placed in the central column of a 3-phase transformer-like iron path (cross section: 30 mm × 30 mm), which acts as a magnetic circuit. The excitation coils are supplied by a power amplifier, controlled by an arbitrary signals generator, as reported in Figure 1a. The central column is cut in two halves—the bottom part is bound with the remaining iron path, while the top half part is connected, through Belleville springs and a load cell, to the Universal Testing Machine (UTM), constituting thus a mechanical chain able to provide the stress to the magnetostrictive sample. The springs are installed because they allow to mitigate the magnetostrictive effect when a constant stress is applied in the mechanical chain. Moreover, the top half part can slide in a square hole made in the upper horizontal arm of the iron part; so that it allows both the stress transfer and the magnetic circuit closing. A Galfenol cylindrical sample (30 mm length and 5 mm diameter) is placed between the two parts of the central column and it is equipped with two strain gauges located on the same external surface but on opposite faces. The measured strain is the average of the two measurement points and any non-uniform stress transfer to the material, due to imperfections, is compensated. A pickup coil is wounded around the sample, while a Hall effect probe (connected to a gaussmeter) is placed perpendicularly in contact with the external surface of the sample, in order to measure the applied magnetic field [25,31]. Both the inputs and outputs are measured by the above mentioned sensors and acquired with a DAQ. The strain, stress and magnetic field are directly measured while the magnetic flux density is measured indirectly. Since in the used experimental setup the cross section of the sample, namely *S* and the pickup coil turns number, namely *N*, do not change, is possible to express the Faraday’s law of induction as:

(1) $$v=-\frac{d\Phi }{dt}=-NS\frac{dB}{dt}$$

where *v* is the measured pickup coil voltage (so the electromotive force, emf), Φ is the linked magnetic flux and *B* the magnetic induction. As a consequence it is possible to write *B* as follows:

(2) $$B=-\frac{1}{NS}\int_{t}v\;dt$$

The integration in Equation (2) is numerically executed in post-processing, in order to obtain the magnetic induction. Finally, being the relation among flux density, magnetization and magnetic field as follows:

(3) $$B=\mu_{0}\left(H+M\right)$$

the magnetization can be obtained.

The characterization of a magnetostrictive materials consists in the measurement of the output when one input changes and the other is kept constant. Therefore, four different types of plots are measurable: $\mu_{0}M-H$ and ε-*H* curves at different constant σ and ΔB-σ and ε-σ curves at different constant *H*. On the other hand, the first and third ones are interesting for EH purposes. At this point it is important to note that the UTM is a device controlling the strain velocity. As a consequence, the set values of the force are applied to the material by a feedback on the force sensor measurement and a null cross-head speed. The magnetostrictive effect counteract the UTM applied stress, by increasing the latter because the crosshead speed control is not sufficient to compensate this variation of force in a short time. To reduce this effect the Belleville springs are introduced in the mechanical chain in order to have a more stable constant applied stress. On the other hand, it should be pointed out that the curves with constant magnetic field are obtained in constant drive excitation coils current, unlike what has been proposed in Reference [32], where the UTM device is directly connected with the sample but a closed loop feedback on the magnetic field is applied.

**Figure 2:**
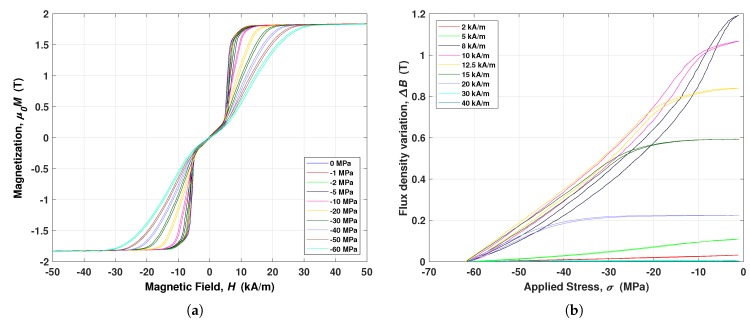
Magneto-mechanical characteristics of SA Galfenol. (**a**) Magnetic characteristics at different compressive applied stress. (**b**) Flux density variation by applying cyclic compressive stress for different magnetic bias conditions (harvesting loops).

Magnetic characteristics, at different constant compressive stress, are plotted in Figure 2a. The curves shown very narrow hysteresis loops, low coercive and saturation field and high saturation magnetization (around 1.7–1.8 T). Furthermore, it is possible to note three distinct regions—the first where the curves are quite overlapped and with a straight slope, the second is characterized by a sudden increasing of slope (so-called “knee bending point”) where the relative permeability is higher than before and, finally, the last region where the material approaches and reaches magnetic saturation. The particular shape of the magnetic cycles, at zero stress, is quite different from common soft ferromagnetic materials, such as non stress annealed (NSA) Galfenol, especially about the presence of the first region. From a phenomenological point of view, this behavior can be ascribed to the annealing process. Indeed, the built-in stress, obtained after the stress annealing process, acts as a pre-stress applied to the annealing-less material (NSA), as reported also in References [24,25]. At this point, it is worth noting that the magnetic curves are quite similar each other with respect to the applied stress. This property, named “*self-similarity*” [26,33,34], has been used to help the material modeling, as shown in the following section. Figure 2b shows the magnetic flux density variation in SA Galfenol, when constants magnetic field and cyclic compressive stress are applied. It should be pointed out that the maximum variation is available at 8 kA/m, that is the optimal bias. Indeed, by comparing this characteristic with the magnetic one, the wider magnetization range between the curves with lower and higher applied stress is obtained after the knee point. Conversely, by increasing the applied constant magnetic bias, the flux variation decreases being negligible at magnetic field larger than 20 kA/m because of the saturation conditions. It is worth to note that the maximum variation is about 1.2 T and the hysteresis is almost negligible.

In conclusion, in this section the usual behaviors of SA Galfenol characteristics are discussed. In particular, some general properties have been recognized and are listed below [34]:

- $\mu_{0}M$-*H* are nonlinear and show narrow hysteresis loop;
- $\mu_{0}M$-*H* show saturation when |H|→∞;
- $\mu_{0}M$ is odd function of *H*;
- the $\mu_{0}M$-*H* cycles are self-similar with respect to the stress;
- regarding $\mu_{0}M$-*H*, if the compressive stress σ increases then the $\mu_{0}M$-*H* cycles drop down.
- regarding ΔB-σ, there is an optimum $H_{opt}$ that makes ΔB largest.

In the next section, these results are exploited to build up an analytical model and an equivalent circuit of a KEH device.

## 3. Modelling

In order to analyze the EH conversion mechanism and to design a reliable energy recovering system, the material modeling or rather the constitutive relations that relate the mechanical and magnetic inputs and outputs, is of great importance [35]. As shown in the previous section, such links are related with strong non-linearity and hysteresis. However, the linear modeling is widely present in literature to describe harvesters, because it allows to obtain analytic expressions concerning the EH phenomenon. On the other hand, it offers acceptable results only when there are small variations of the inputs. These conditions are quite unreal for standard applications. Indeed, when such approximation is adopted, notable errors in outputs, with respect to the magnetic bias, are obtained [36,37,38].

For EH purposes, the constitutive relations should consider the losses, due to hysteresis or eddy currents. The latter process, for suitable small dimensions of the active sample with respect to the frequency ranges, could be neglected. Conversely, hysteresis loop shape does not depend on the input rate. Moreover, its area represents a loss energy density and, under periodic input excitation, the dissipated power is proportional to the frequency. On the other hand, when Galfenol is considered, the coercive field is very small and the hysteresis loop is narrow. Then, the hysteresis losses can be not taken into account while maintaining the model accuracy. This implicates the construction of several memory-less nonlinear models, to describe the magnetostrictive relationships [21,39]. However, none of them have been adopted in the complete modeling of a KEH device, as described hereafter. In particular, in the following a nonlinear fully-coupled model for SA Galfenol and an equivalent circuit of a single rod KEH device are shown.

### 3.1. Analytical Model of SA Galfenol

The modeling of a cylinder presented here is based on the following hypothesis:

- all mechanical input and fields are parallel to the cylinder axis;
- any transformations is isothermal;
- hysteresis phenomena is negligible;

then, the *Gibbs* free energy density can be considered as [25,26,34,40,41]:

(4) $$G\left(\sigma ,H\right)=\frac{\sigma^{2}}{2E}+\frac{\mu_{0}H^{2}}{2}+\Psi \left(\sigma ,H\right)$$

where *E* and $\mu_{0}$ are, respectively, the Young’s Modulus and the vacuum magnetic permeability.

The first two terms on the right hand side of Equation (4) constitute pure linear elastic and magnetic energy contributions, respectively. While the third term is the Gibbs free energy contribution due to the magneto-mechanical coupling of magnetostrictive materials and the function Ψ(σ,H) is properly determined in accordance with the material characteristics. *H* and σ are the state variables, while ε=ε(σ,H) and B=B(σ,H) are the state functions. It is worth to note that if hysteresis is considered, then the memory state is an additional state variable [40]. Conversely, by neglecting the hysteresis, any transformation inside the material can be considered as *lossless*. From a thermodynamic point of view, this brings to the following constrain [39,42]:

(5) $${\left.\frac{\partial \varepsilon }{\partial H}\right|}_{\sigma }={\left.\frac{\partial B}{\partial \sigma }\right|}_{H}$$

where the variables σ and *H* as suffix indicate that the derivatives are taken by assuming those variables as constants.

The Gibbs free energy, previously identified in Equation (4), is a state function, that is, it is a quantity that depends uniquely on the state of a system, since it admits an exact differential in a close range of an equilibrium state and, therefore, does not depend on previous thermodynamic transformations. At this stage, the conditions expressed in Equation (5) allow to relate the σ and *H* state variables within the corresponding ε and *B* state functions as follows:

(6) $$\left\{\begin{array}{cc} \varepsilon & ={\left.\frac{\partial G}{\partial \sigma }\right|}_{H}\\ B & ={\left.\frac{\partial G}{\partial H}\right|}_{\sigma } \end{array}\right.$$

The relations expressed in Equations (4) and (6) indicate that the determination of the function Ψ(σ,H) allows to obtain the nonlinear model. It is assumed here that the function Ψ(σ,H) is expressed as [26,34,40,41]:

(7) Ψ(σ,H)=f(σ)·u(z)

where z=z(σ,H). The functions f(σ), u(z) and z(σ,H) must be suitably determined, according to the material characteristics, in order to mimic the main physical behaviors of the material, as saturation effect and magnetization dependence by the stress [34,41]. Consequently, the Gibbs free energy is:

(8) $$G\left(\sigma ,H\right)=\frac{\sigma^{2}}{2E}+\frac{\mu_{0}H^{2}}{2}+f\left(\sigma \right)\;\cdot \;u\left(z\right)$$

By deriving G(σ,H) once respect to σ at constant *H* and once respect to *H* at constant σ, as in the relations expressed in Equation (6), it can be obtained ε and *B*, respectively, in the nonlinear case [26]:

(9) $$\left\{\begin{array}{cc} \varepsilon & ={\left.\frac{\partial G}{\partial \sigma }\right|}_{H}=\frac{\sigma }{E}+f^{\prime }\left(\sigma \right)\;\cdot \;u\left(z\right)+f\left(\sigma \right)\;\cdot \;u^{\prime }\left(z\right)\;\cdot \;\frac{\partial z}{\partial \sigma }\\ B & ={\left.\frac{\partial G}{\partial H}\right|}_{\sigma }=\mu_{0}H+f\left(\sigma \right)\;\cdot \;u^{\prime }\left(z\right)\;\cdot \;\frac{\partial z}{\partial H} \end{array}\right.$$

where $f^{\prime }\left(\sigma \right)$ and $u^{\prime }\left(z\right)$ represent the derivatives of the functions f(σ) and u(z) respectively, that is,

$$f^{\prime }\left(\sigma \right)=\frac{df(\sigma )}{d\sigma },\;u^{\prime }\left(z\right)=\frac{du(z)}{dz}$$

By starting from the general expressions of Equation (9) and by using the assumption of z=H/f(σ), in order to take into account the dependence of the magnetic response from the applied stress [40,41], it is possible to re-arrange the general nonlinear model as follows [34]:

(10) $$\left\{\begin{array}{cc} \varepsilon & ={\left.\frac{\partial G}{\partial \sigma }\right|}_{H}=\frac{\sigma }{E}-f^{\prime }\left(\sigma \right)\;\cdot \;\left[z\;\cdot \;u^{\prime }\left(z\right)-u\left(z\right)\right]\\ B & ={\left.\frac{\partial G}{\partial H}\right|}_{\sigma }=\mu_{0}H+u^{\prime }\left(z\right) \end{array}\right.$$

It should be noted that the second of Equation (10) implies that $u^{\prime }\left(z\right)$ constitutes the magnetic polarization M(H,σ) of the material. Then, the terms $u^{\prime }\left(z\right)$, its integral u(z), f(σ) and *z* have to be chosen to fit Equation (10) for the adopted material. To this scope, for SA Galfenol, it has been assumed [26]:

(11) $$M\left(H,\sigma \right)=u^{\prime }\left(z\right)=\frac{\alpha \;\cdot \;z}{\beta +z^{4}}+M_{s}\;\cdot \;\tanh\left(z\right)$$

(12) $$f\left(\sigma \right)=\gamma \;\cdot \;\left(\sigma +\sigma_{b}\right)$$

(13) $$z=\frac{H}{f(\sigma )}=\frac{H}{\gamma \;\cdot \;(\sigma +\sigma_{b})}$$

where $M_{s}$ is the magnetic saturation, $\sigma_{b}$ is the built-in stress due to the stress annealing process, while α, β and γ are some model’s parameters.

The Equations (11)–(13) are found on a phenomenological base, with the aim to reproduce the magnetic characteristic of SA Galfenol discussed in Section 2. In particular, the three distinct regions of the magnetic induction at constant stress and its slope varying by increasing the mechanical stress are taken into account. The function expressed by the Equation (11) is odd and consists of two terms:

- a fractional fourth order function in the *z* variable, which describes SA Galfenol behavior at low magnetic field applied and the above mentioned kinking phenomena;
- an hyperbolic function able to mimic the response for increasing magnetic field and saturation.

Finally, as described by the first equations of the sets (9) and (10), the strain ε(H,σ) is derived starting from Equations (11)–(13). Then, it follows [26]:

(14) $$\varepsilon \left(H,\sigma \right)=\frac{\sigma }{E}-\gamma \;\cdot \;\left\{z\;\cdot \;\left[\frac{\alpha \;\cdot \;z}{\beta +z^{4}}+M_{s}\;\cdot \;\tanh\left(z\right)\right]-\left[\frac{\alpha }{2\sqrt{\beta }}\;\cdot \;\arctan\left(\frac{z^{2}}{\sqrt{\beta }}\right)+M_{s}\;\cdot \;\ln\left(\cosh\left(z\right)\right)\right]\right\}$$

The parameters $M_{s}$ and $\sigma_{b}$ can be directly obtained by a comparison with experimental data, while the remaining parameters are determined by using a standard minimization technique. All the steps about the identification procedure are described in Reference [26]. More in detail, the parameters $M_{s}$ and $\sigma_{b}$ are correlated to the *M*-*H* curves at different constant stresses, that are shown in Figure 2a. From these measurements the magnetic saturation can be assumed to be $M_{s}=1.72$ T. By examining the above mentioned curves, by increasing the stress at the same magnetic field, the magnetic polarization *M* decreases. Moreover, if the magnetic field is enhanced, the magnetic polarization also increases. Consequently, the material gets to be magnetically harder when larger compressive stress are applied and, furthermore, all curves shown a similar profile, as discussed in Section 2. Consequently, it is said that the curves are self-similar [26,33,34,43] and, by looking for a suitable scaling methodology between input variables (*H*, σ), all curves almost drop into a single one. In this study, the condition of self-similarity is attained by assuming the magnetic field *H* divided by the complete stress applied to the Galfenol rod, that is, $\sigma +\sigma_{b}$, where $\sigma_{b}$ is the built-in stress and σ the external applied stress. Since the built-in stress is a proper characteristic of the material, it is constant for all curves and can be achieved as consequence. Then, the value of $\sigma_{b}$ is matched in such a way that the upshot curves of the *M* vs. $H/(\sigma +\sigma_{b})$ are overlapped. For the considered SA Galfenol sample, $\sigma_{b}$ is found to be −50 MPa such that the curves of *M* vs. $H/(\sigma +\sigma_{b})$ present a matching profile as shown in Reference [26]. This value agrees with what is found in the literature [27], where the built-in stress values are obtained with an energy-based model minimization method and from manufacturer [30].

**Figure 3:**
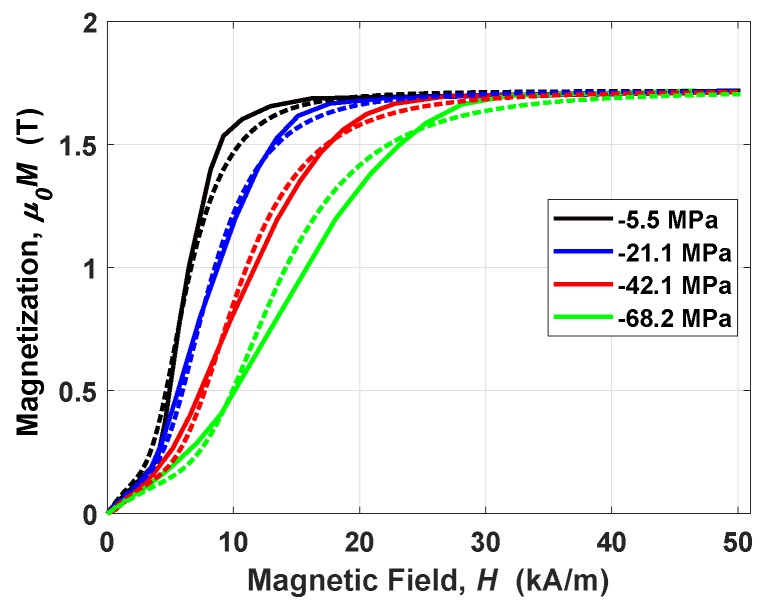
Magnetic characteristic (first quarter) of SA Galfenol, at different compressive stress—comparison between measurements (solid line) and simulation of Equation (11) (dashed line).

The other three parameters in Equations (11)–(13) have been achieved with a nonlinear least mean squares method applied between measurements and the nonlinear model. The procedure, shown in Reference [26], is to find α, β and γ such that the error between model and measurements is minimum. The method gave as results α=−1.1233 T, β=0.8415 and $\gamma =-9.7927\times 10^{-5}$ T^−1^, with a normalized residual of 0.0938. The comparison among the simulated magnetic characteristics and the measured ones is reported in Figure 3. The agreement is quite remarkable. It is worth to highlight that the curves of Figure 3 have been achieved by starting from the cycles of Figure 2a. In particular, the descending and ascending branches of the cycles are averaged point by point into the hysteresis-less curves.

In conclusion, the Equations (11)–(14) constitute a fully coupled nonlinear model, capable to mimic the typical behavior of SA Galfenol.

### 3.2. Three-Port Equivalent Circuit of a Single Rod KEH Device

In a KEH devices based on magnetostrictive materials, mechanical, magnetic and electric quantities are involved and coupled together [44]. Therefore, it may be difficult to study and design the different parts without a model that takes into account all the interactions. Then, an equivalent circuit, where the electrical components are related with the above-mentioned quantities, could be a handy tool able to simulate the working conditions of the KEH with a circuit simulator, like LTspice [45].

**Figure 4:**
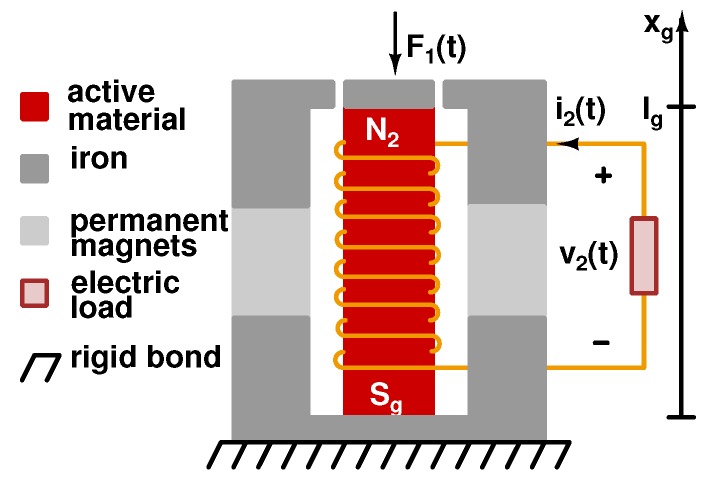
2-D sketch of a force driven magnetostrictive KEH concept device and its main elements.

The nonlinear model previously developed has been used in this section to build up an equivalent lumped parameters circuit of a KEH device with a single rod of SA Galfenol. In such a way, it is possible to simulate a force-driven harvester in different conditions. In particular, the KEH device belongs to the force driven category, that is, the force source is directly put in contact with the active material, along the longitudinal axis, as reported in Figure 4.

In spite of the arrangements, the main elements of a force driven KEH device are cataloged in:

- the active material, in this case SA Galfenol;
- a mechanical frame devoted to transfer the force to the active material;
- the magnetic circuit providing the magnetic bias to improve the harvested power;
- the coil, generally wounded around the active material, in order to exploit the Villari effect and Faraday’s law.

Then, by considering the sketch of the KEH concept device shown in Figure 4, the Galfenol rod, with length $l_{g}$ and a cross section $S_{g}$, is located in the central part. The iron frame has the twofold role as mechanical frame and magnetic circuit, where $l_{fe}$ is the center line length and $S_{fe}$ is the cross section. From the topside of the iron frame, through an air gap, a force $F_{1}\left(t\right)$ is applied to the sample. The iron structure hosts the PMs with length $l_{m}$ and cross section $S_{m}$. Finally, the conversion of vibrational-mechanical energy into electrical one is possible by exploiting both the inverse magnetostriction effect and Faraday’s law, by means of a $N_{2}$-turn coil placed around the active rod. The analysis presented in the following has been performed under these assumptions [26,34,41]:

1. all the fields are coaxial and directed along the magnetostrictive material axis, in such a way to have a scalar representation of the constitutive relationships;
2. the frequency of the applied force is small enough in such a way that it can be neglected the mechanical propagation phenomena in the rod. As a consequence, any mechanical element can be modeled as lumped mass, spring, damper. Moreover, vibrations are much lower than the mechanical resonance of the structure.
3. the previous point implies that the stress is assumed uniformly distributed along the rod axis;
4. the electromagnetic field propagation is neglected, then the electric load is considered as a lumped element;
5. the magnetic circuit is assumed to be a flux tube for the magnetic induction;
6. eddy currents are negligible and magnetic circuit theory can be used to model the magnetic part.

The complete system can be now modeled through circuit tools giving an explicit analysis of the harvester. The equivalent three-port circuit, through particular analogies, relates to each other all the mechanical, magnetic and electric quantities of the global system, such as the force, the output voltage and the magnetization of PM. Moreover, all the possible external elements involved in the harvester can be suitably added to the model, including the mechanical system model, the biasing model and the electric load. At this point it is worth to note that, from the assumptions previously taken into account, the equivalent circuit has lumped elements [21,26,34,41,46,47]. Then, regarding the magnetostrictive sample, it is possible to express the applied stress as $\sigma =F_{1}/S_{g}$, the strain as $\varepsilon =x_{g}/l_{g}$ and the magnetic flux as $\phi_{g}=B_{g}\;S_{g}$, where $B_{g}$ is the magnetic flux in the rod and $x_{g}$ is the magnetostrictive rod tip displacement.

Let’s start with the representation of the active rod. Let’s consider the set of Equation (10), which is rewritten in order to make them more appropriate for equivalent circuit modeling. By following the same line of reasoning adopted in References [26,34,41], the first of the Equation (10) is multiplied by $l_{g}$ and the second equation by $S_{g}$, as follows:

(15) $$\left\{\begin{array}{cc} x_{g} & =\frac{F_{1}}{\xi }+l_{g}\;\cdot \;g\left(F_{1},H_{g}\right)\\ \phi_{g} & =\mu_{0}\;H_{g}\;S_{g}+S_{g}\;\cdot \;m\left(F_{1},H_{g}\right) \end{array}\right.$$

where:

- $\xi =E\;S_{g}/l_{g}$ is the mechanical stiffness of the magnetostrictive sample;
- $g\left(F_{1},H_{g}\right)=-f^{\prime }\left(\sigma \right)\;\cdot \;\left[z\;\cdot \;u^{\prime }\left(z\right)-u\left(z\right)\right]$;
- $\phi_{g}$ and $H_{g}$ are, respectively, the magnetic flux and the magnetic field in the magnetostrictive rod;
- $m\left(F_{1},H_{g}\right)=u^{\prime }\left(z\right)$.

In the second equation of the set of Equation (15), the term $\mu_{0}\;H_{g}\;S_{g}$ can be rewritten as $l_{g}\;H_{g}/R_{g}$, where $R_{g}=l_{g}/\left(\mu_{0}\;S_{g}\right)$ represents the linear contribution of the total *reluctance* of the magnetoelastic rod. Indeed, generally, the magnetic permeability of a magnetostrictive material, such as Galfenol, can be split into two parts—obviously a nonlinear part, represented in this case by the model and a linear contribution represented by $R_{g}$. The system of Equation (15) can be arranged as follows:

(16) $$\left\{\begin{array}{cc} F_{1} & =\xi \;x_{g}-l_{g}\;\xi \;\cdot \;g\left(F_{1},H_{g}\right)\\ H_{g}\;l_{g} & =R_{g}\;\phi_{g}-R_{g}\;S_{g}\;\cdot \;m\left(F_{1},H_{g}\right) \end{array}\right.$$

The following analogies between the mechanical and electric quantities are adopted to convert the mechanical equation in the first of the set of Equation (16), into the electrical equivalent. In particular:

- the force corresponds to a voltage, $F_{1}\left(t\right)⟺v_{1}$ ;
- the rod tip velocity corresponds to a current, ${\dot{x}}_{g}\left(t\right)⟺i_{1}$ .

The following set of equations are consequently achieved:

(17) $$\left\{\begin{array}{cc} v_{1}\left(t\right) & =\xi \int_{t}i_{1}\;dt-l_{g}\;\xi \;\cdot \;g\left(v_{1},H_{g}\right)\\ H_{g}\;l_{g} & =R_{g}\;\phi_{g}-R_{g}\;S_{g}\;\cdot \;m\left(v_{1},H_{g}\right) \end{array}\right.$$

For the second of the set of Equation (17), that is, the magnetic part and by applying magnetic circuit theory, the quantities $H_{g}\;l_{g}$ corresponds to a magnetic voltage. As a consequence, the analogies among magnetic voltage and electrical one can be made, that are:

- $H_{g}\;l_{g}\Leftrightarrow v_{g}$ ;
- the reluctance $R_{g}$ is modeled by a resistance $R_{g}$ ;
- $i_{g}$ is the flux $\phi_{g}$, which flows in the reluctance rod $R_{g}$ .

(18) $$\left\{\begin{array}{cc} v_{1}\left(t\right) & =\xi \int_{t}i_{1}\;dt-l_{g}\;\xi \;\cdot \;g\left(v_{1},\frac{v_{g}}{l_{g}}\right)\\ v_{g} & =R_{g}\;i_{g}-R_{g}\;S_{g}\;\cdot \;m\left(v_{1},\frac{v_{g}}{l_{g}}\right) \end{array}\right.$$

**Figure 5:**
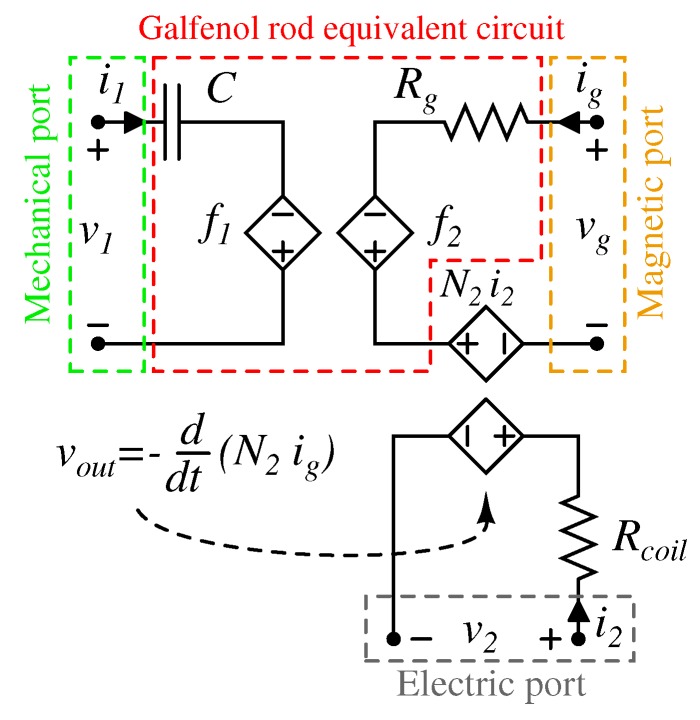
Equivalent three-port nonlinear model circuit of a Galfenol rod. In the first port, voltages and currents correspond to forces and velocities, respectively, while in the second port are magnetic voltages and flux. Furthermore, voltages and currents of the third port correspond to real quantities delivered to the load. The nonlinear functions are: $f_{1}=l_{g}\;\xi \;\cdot \;g\left(v_{1},\frac{v_{g}}{l_{g}}\right)$ and $f_{2}=R_{g}\;S_{g}\;\cdot \;m\left(v_{1},\frac{v_{g}}{l_{g}}\right)$.

The previous set of Equation (18) constitutes the loop Kirchhoff’s voltage law for a well-posed two-port circuit, which is similar to the one reported in Reference [41]. The first port is composed by a voltage source, that is, the external applied force, with a series capacitance, $C=1/\xi =l_{g}/\left(E\;S_{g}\right)$ and a nonlinear dependent voltage source, which represents the nonlinear magnetostrictive response of the rod. The second equation can be modeled as a voltage source, that is, the source of magnetic bias, with a series resistance, which represents the linear reluctance and a nonlinear dependent voltage source that takes into account the nonlinear magnetic response of rod, as shown in Figure 5.

The last missing part is constituted by the electric energy conversion, which is not yet expressed in Equation (18). In particular, the output voltage across the pickup coil, wounded around the active material, is due from the exploitation of the Villari effect and the Faraday’s law. The first is considered in the $m(v_{1},\frac{v_{g}}{l_{g}})$ function of the nonlinear model, while the latter reads:

(19) $$v_{out}\left(t\right)=-\frac{d}{dt}\Phi_{g}\left(t\right)$$

where $\Phi_{g}\left(t\right)=N_{2}\;\phi_{g}$ is flux linkage in $N_{2}$-turn pickup coil.

In the equivalent circuit, the coil flux linkage is modeled by $N_{2}\;i_{g}$; then when a current flows in the pickup coil, this current will influence the total flux in the magnetostrictive element by the transformer effect. As a consequence, a voltage source, corresponding to the $N_{2}\;i_{2}$ of the pickup coil circuit, is added in series in the second port of Figure 5. Finally, a third port, representing the electric side, is added in the circuit model where $R_{coil}$ is the resistance of the pickup coil, $i_{2}$ is the current which flows in the pickup coil and $v_{2}$ is the output voltage. In conclusion, Figure 5 reports the *general* three port circuit which represents the bare magnetostrictive material rod with a $N_{2}$ winding.

The set of Equation (18) and the above mentioned different analogies point out that, with the aim to model a whole device, further lumped circuit elements can be inserted now to the three ports. For example, by considering the concept device of Figure 4, if the harvester undergoes a vibrating force generator and the Galfenol mass $m_{g}$ is considered then these can be simply solved by connecting the series of a voltage generator $v_{\mathrm{force}}$ and a induct or $m_{g}$ to the first port. While a viscous friction is represented by a resistor:

(20) $$\begin{array}{cc} F=m\frac{d^{2}x}{dt^{2}}\; & ⟺\;v=m\frac{di}{dt}\\ F=r\frac{dx}{dt}\; & ⟺\;v=r\;i\; \end{array}$$

**Figure 6:**
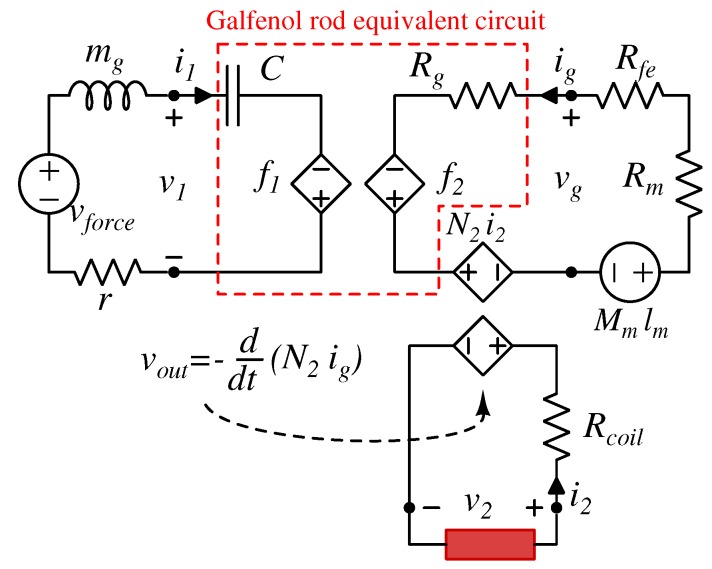
Equivalent three port nonlinear model circuit of the concept device in Figure 4.

Moreover, by considering the magnetic circuit theory, the iron frame and the PM are represented by a reluctance $R_{\mathrm{fe}}$ and the series of a reluctance $R_{g}$ and a magnetomotive force $M_{m}l_{m}$, as reported in Figure 6 where also the generic electric load is connected to the electric port.

## 4. Characterizations and Simulations of the Three Rod Device

The three-port equivalent circuit has been validated on a real KEH device based on a single rod of SA Galfenol, as shown in Reference [26]. More in details, the model and the equivalent circuit are able to receive a force profile as input and to provide an output voltage with good accuracy, with the exception of very low and high biasing fields, that are not effective for EH aims. Then, in this section, the effectiveness of the equivalent circuit is tested on a KEH system, conceived for a realistic application, which has been proposed in References [16,17].

The core of the system, consisting in a three-rods SA Galfenol KEH device, is presented, characterized and modeled. The device is enclosed within two steel plates, with a height of 6 mm and diameter of 52 mm, that connect three Galfenol rods, with a length of 21 mm and diameter of 6 mm, 120° spaced and 18 mm far from the center. The rods have pilot pins (6 mm height) entering in the disks. Furthermore, the top plate hosts a steel sphere (10 mm diameter) to provide a single contact point with the external force source, in order to equally transfer the stress to each rod. A column of neodymium disk PMs is placed in the center to provide a magnetic bias to the Galfenol rods. Indeed, the two steel plates have a twofold purpose—they are a low reluctance path for the PMs flux and they grant the mechanical stress transfer to the active elements.

**Figure 7:**
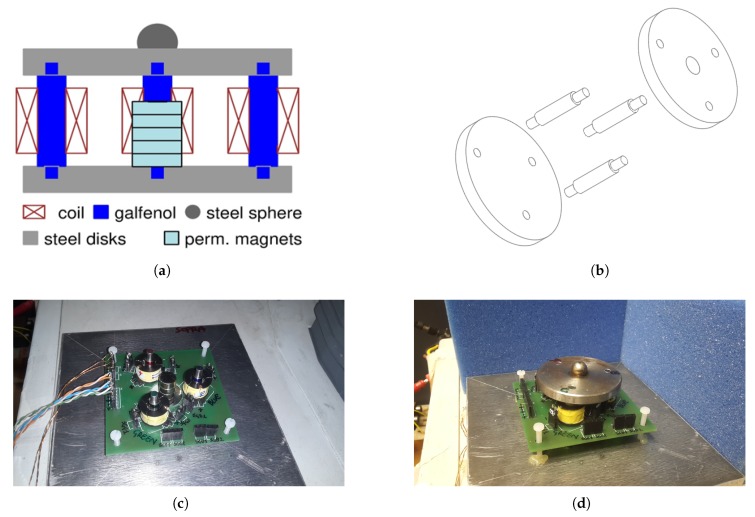
KEH device based on three Galfenol rods. PMs are inserted to provide the magnetic bias. (**a**) 2-D sketch. (**b**) 3-D sketch. (**c**) Photo of the PCB, PMs and Galfenol rods during installation. (**d**) Photo of the whole 3-rod kinetic energy harvesting (KEH).

A PCB (70 mm × 70 mm and 3 mm thick), shown in Figure 7c,d has been designed and carried out to have reliable electrical connections for three 2000-turn pickup coils. Figure 7 shows the three-rods Galfenol KEH device developed. In particular, in the Figure 7a,b two drafts regarding the mechanical connections are depicted, while in Figure 7c, two photos of the device are shown.

The proposed three-rods KEH system can be used with different coils connection, PMs configurations and force types, then permitting different electrical, magnetic and mechanical operative conditions. Consequently, design work is important to identify the system’s parameters, such as the electric load, the pickup coils connections and the typology of magnets, in order to optimize the harvested energy. Periodic compressive forces, at three different low-frequencies, have been applied by the UTM. Then, discs PM (20 mm length) with three different diameters (10, 15 and 20 mm) have been tested. In addition to the three-rods KEH device’s characterization, a whole equivalent circuit has been performed and simulated. In such a way, the experimental data and the simulations can be compared in order to estimate the circuital model goodness.

**Figure 8:**
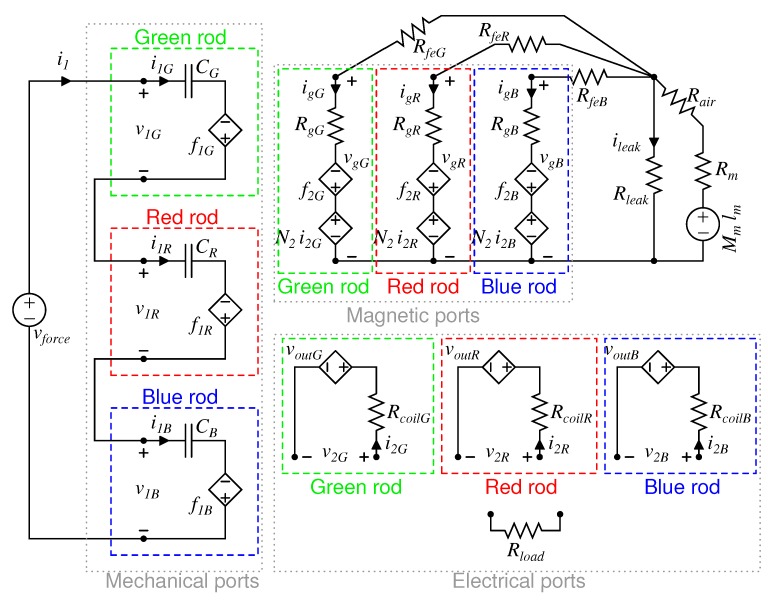
Equivalent three port nonlinear model circuit of the three-rods Galfenol KEH. The mechanical ports are connected in series because the universal testing machine (UTM) speed is the same for each rod, while the electrical ports can be connected together in series or parallel on a resistive load.

In the light of results shown in Section 3.2, the equivalent circuit of the three-rods KEH device is depicted in Figure 8. On the behalf of clarity, the three rods are distinguished with different colors—green, red and blue. The three-port equivalent circuit of each rod has been highlighted by the corresponding color. The mechanical ports are connected in series because the force applied to the steel sphere is divided to each rod. The applied force is represented by a voltage generator. On the magnetic side, each port is connected, through its steel reluctance ($R_{fe}$), to the neodymium magnets. As for the single rod case, the equivalent circuit of the PMs stack is a real voltage generator. The PM height is 1 mm lower than the rod ones, then the corresponding air gap reluctance, $R_{air}$, is added in series. By increasing the diameter of PMs, the value $M_{m}l_{m}$ remains constant, while the reluctance $R_{m}$ decreases. Finally, $R_{leak}$ represents any leakage of the magnetic flux. It has been placed in parallel with $R_{air}$ and PM circuit series because, for this device, it is reasonable to consider the leakage flux closing from the top to the bottom disc. Details of the above circuit elements are reported in Appendix A.

**Figure 9:**
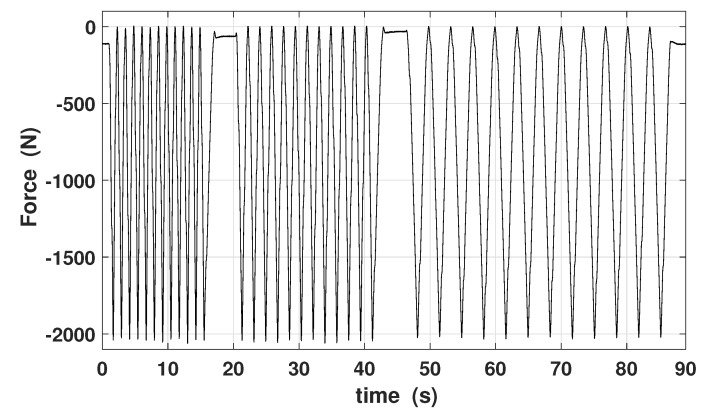
Force profile applied to the 3-rod KEH device. It consists in ten compressive cycles from 0 to −2000 N at different UTM speeds—150, 100, 50 mm/min.

Low frequency mechanical stress variations can be generated by the UTM, producing a compressive force with a period larger than 1 s. A load cell is used to measure the instantaneous force applied to the device. The applied force is constituted by the same profiles but repeated with different time rates and each profile consists in ten complete cycles. Consequently, all profiles are a compressive force varying from 0 up to −2000 N, although with three distinct UTM crosshead speeds, that are of 150, 100 and 50 mm/min respectively, corresponding in frequencies of about 0.8, 0.55 and 0.3 Hz. The measured stress profile applied to the three-rods KEH device is plotted in Figure 9. The force measurement is acquired and can be exploited as the input equivalent force generator ($v_{force}$), in order to make simulations in LTspice.

**Figure 10:**
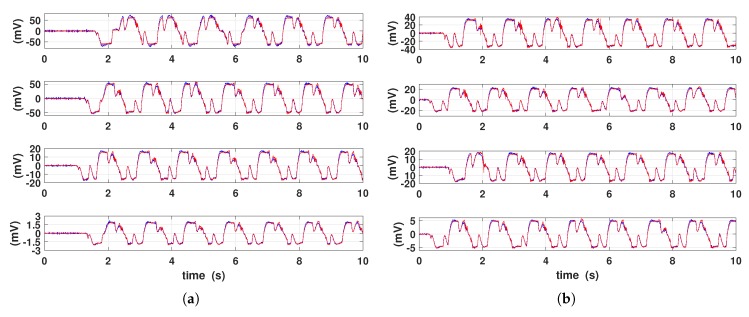
Measured (color red) and simulated (color blue) output voltages. 15 mm diameter PM and 150 mm/min strain velocity are applied. (**a**) Coils connected in series on 10 k, 525, 100 and 10 Ω (from top to bottom pane). (**b**) Coils connected in parallel on 10 k, 100, 58 and 10 Ω (from top to bottom pane).

The time-profiles of measured and simulated output voltages for different coils connection are shown in Figure 10. For the sake of shortness, only the 15 mm diameter PM and 150 mm/min strain velocity case has been reported for different resistive loads. The equivalent circuit is able to mimic quite well the shape of the output voltage. Furthermore, as expected, the measured voltage increases for larger electric load values, both for series and parallel pickup coil connections.

**Figure 11:**
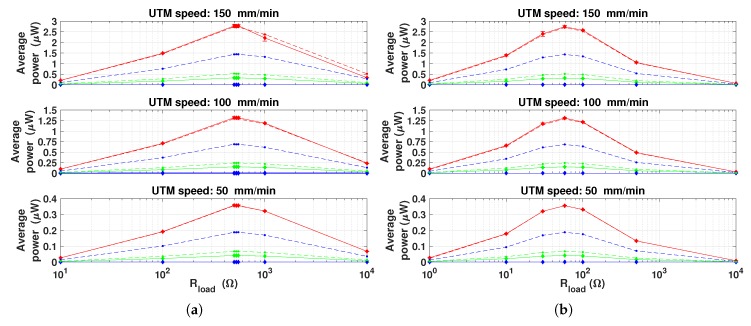
Comparison between the average measured (diamonds with solid line) and simulated (dots with dashed line) power vs. the applied electric load. PMs with different diameters are considered—10 (green), 15 (red) and 20 mm (blue). (**a**) Coils connected in series. (**b**) Coils connected in parallel.

**Figure 12:**
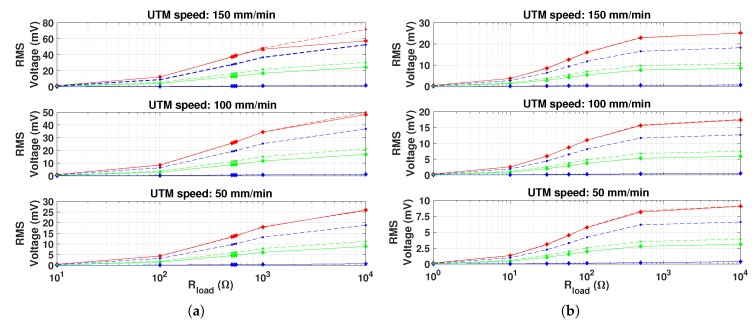
Comparison between the RMS measured (diamonds with solid line) and simulated (dots with dashed line) voltage vs. the applied electric load. PMs with different diameters are considered—10 (green), 15 (red) and 20 mm (blue). (**a**) Coils connected in series. (**b**) Coils connected in parallel.

**Figure 13:**
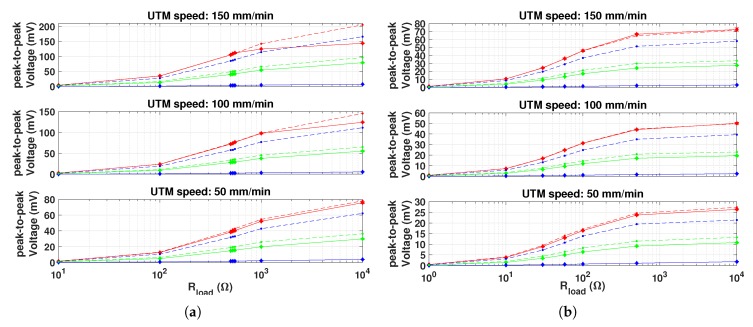
Comparison between the peak-to-peak measured (diamonds with solid line) and simulated (dots with dashed line) voltage vs. the applied electric load. PMs with different diameters are considered—10 (green), 15 (red) and 20 mm (blue). (**a**) Coils connected in series. (**b**) Coils connected in parallel.

The measured and simulated average power, RMS and peak-to-peak voltage with respect to different electric loads, magnets diameter and UTM speeds, are shown in Figure 11, Figure 12 and Figure 13, respectively. Each measurement point is obtained by averaging over ten cycles and by calculating the standard deviation, that is represented with error bars, in order to define the confidence interval measurements.

About the performance of the device, the maximum power is obtained when the load is equal to the total coil resistance. Power peaks are present around $R_{load}$ = 525 Ω, in case of coils connected in series and around $R_{load}$ = 58 Ω, in case of parallel connection. Indeed, these two values represent, respectively, the total equivalent coil resistance (the single coil resistance is 175 Ω, as reported also in Appendix A). This is in agreement of the optimum energy transfer theorem.

The input frequency strongly affects the harvested power. Indeed, it is noticeable that by doubling the force input frequency, the average power quadruples. Moreover, the peaks average power both for series and parallel connections are equal, when the same force profile and optimal resistive load are applied. In other terms, from the energy conversion point of view, when optimal electric load condition is applied, the two connections are equivalent [17]. By increasing the force frequency and the output load, the RMS and peak-to-peak voltages also increase [16,17], as a explicit consequence of Ohm’s law and Faraday’s law. As expected, the harvested power is depending on the diameter of PM used as they provide the magnetic bias. In particular, the 15 mm diameter case gave the largest power values, about 8 times larger than the 10 mm diameter case. The curves referred to the 20 mm diameter case show that the average power, RMS and peak-to-peak voltage are very low. This may suggest that the magnetic saturation condition occurs in the Galfenol rods.

The comparison between measurements and simulations shows that the circuital model is able to predict the average power with an error lower than 5% when optimal PM is considered. On the other hand, errors around 60% and larger than 100% occur when 10 mm and 20 mm diameter PM, respectively, are applied. In the latter case, magnets and Galfenol rods are very close each other, then magnetic leakage increases. As a consequence, the comparison among measurements and simulations suggests that, due to lumped parameters, the model is not able to reproduce these conditions.

Finally, the power harvested by the KEH device under study is comparable with other EH device presented in literature. For example, the measured output power in Reference [48] is 450 mW with 60 Hz frequency of the applied force, while it is about 16 mW at 100 Hz in Reference [49] and 0.1 mW at 5 Hz for the device presented in Reference [50]. In order to make a comparison, by considering that in first approximation the output power scales with the square of the input frequency, it is possible to estimate a KEH device’s output powers of about 0.75 mW at 5 Hz, 11 mW at 60 Hz and 30 mW at 100 Hz, that are comparable by considering the volume of active material.

## 5. Conclusions

In this work, the characterization and modeling of a KEH device based on Galfenol has been concerned. With this aim, a nonlinear model of SA Galfenol have been adopted and its parameters have been determined by using the measured magnetostrictives curves. The model takes into account the most relevant experimental behaviors, that is, nonlinearity, the magneto-mechanical coupling and saturation effects. Because of the mechanical, magnetic and electric quantities involved in a KEH device, a three-port equivalent circuit of a concept device based on a single Galfenol rod is reported and described. In sight on the results obtained, a more complex KEH system based on three Galfenol rods, has been experimentally characterized and modeled. The characterization has been carried out by applying different compressive force profiles, electric loads and permanent magnets. As expected, the input force frequency and the magnets configuration strongly affect the output voltage and power, while an optimal resistive load, corresponding to the total equivalent coil resistance, is needed to extract the maximum power (about 3 µW at 0.8 Hz of input force frequency and optimal bias and electric load). From the energy conversion point of view, no differences have been found among series and parallel coils connection. Furthermore, the comparison among experimental data and simulations have confirmed the ability of the circuital model to predict the output voltage and harvested power for different loads and pickup coils connection when the optimal bias condition is concerned.

## Figures and Tables

**Table 1:** Some properties of Galfenol [30].

Standard Composition	Fe_81.6_Ga_18.4_
**Mechanical Properties**	
Density	7800 kg/m^3^
Young’s Modulus at constant I	40–60 GPa
Young’s Modulus at constant V	60–80 GPa
Bulk Modulus	125 GPa
Speed of Sound	2265–2775 m/s
Tensile Strength	350 MPa
Fatigue Strength	75 MPa fully reversed
Minimum Laminate Thickness	0.25 mm
**Thermal Properties**	
Thermal expansion coefficient	11 ppm/°C at 25 °C
Thermal Conductivity	15–20 W/(mK) at 25 °C
Melting Point	1450 °C
**Electrical Properties**	
Resistivity	85 × 10^−8^ Ωm
Curie Temperature	670 °C
**Magnetostrictive Properties**	
Strain (estimated linear)	200–250 ppm
Energy Density	0.3–0.6 kJ/m^3^
Piezomagnetic Constant, d_33_	20–30 nm/A
**Magnetomechanical Properties**	
Coupling Factor	0.6–0.7
**Magnetic Properties**	
Relative Permeability	75–100
Saturating Magnetic Field	8–20 kA/m
Coercivity, H_*c*_	∼800 A/m
Hysteresis (major loop)	1000 J/m^3^
Saturation Flux Density	1.5–1.6 T

## Abbreviations

The following abbreviations are used in this manuscript:

ICT	Information and Communications Technology
PDA	Personal Digital Assistant
WSN	Wireless Sensors Network
SHM	Structural Health Monitoring
EH	Energy Harvesting
IoT	Internet of Things
KEH	Kinetic Energy Harvesting
NSA	Non Stress Annealed
SA	Stress Annealed
PCB	Printed Circuit Board
PM	Permanent Magnet
RMS	Root Mean Square

## Appendix A. Identification Procedure of the Three-Rods Galfenol Keh Device Equivalent Circuit Parameters

This appendix is devoted to present more in detail and give hints on the identification of the equivalent circuit of the three-rods device, represented in Figure 8.

By considering x∈{G,R,B}, the parameters are: $M_{s}$, $\sigma_{b}$, α, β, γ, *E*, $C_{x}$, $f_{1x}$, $f_{2x}$, $R_{fex}$, $R_{leak}$, $R_{air}$, $R_{m}$, $M_{m}$, $l_{m}$, $R_{gx}$, $N_{2}$, $v_{outx}$, $R_{coilx}$, $R_{load}$.

In the following a list of the parameter’s values is presented:

1. $M_{s}$, $\sigma_{b}$, α, β, γ, are related to the proposed nonlinear model for SA Galfenol and are detailed in Section 3.1.
2. $C_{G}=C_{R}=C_{B}=1/\xi =l_{g}/\left(ES_{g}\right)$, are equals because same rods have been used.
3. $l_{g}=21$ mm and $S_{g}=\pi {\left(3\right)}^{2}$ mm^2^ are geometric quantities of the employed rods, so directly measurable.
4. E=60 GPa is the Galfenol Young Modulus, which can be determined from the material datasheet.
5. $f_{1G}=l_{g}\;\xi \;\cdot \;g\left(v_{1G},\frac{v_{gG}}{l_{g}}\right)$, $f_{1R}=l_{g}\;\xi \;\cdot \;g\left(v_{1R},\frac{v_{gR}}{l_{g}}\right)$ and $f_{1B}=l_{g}\;\xi \;\cdot \;g\left(v_{1B},\frac{v_{gB}}{l_{g}}\right)$ are the nonlinear dependent voltage sources on the mechanical ports.
6. $f_{2G}=R_{g}\;S_{g}\;\cdot \;m\left(v_{1G},\frac{v_{gG}}{l_{g}}\right)$, $f_{2R}=R_{g}\;S_{g}\;\cdot \;m\left(v_{1R},\frac{v_{gR}}{l_{g}}\right)$ and $f_{2B}=R_{g}\;S_{g}\;\cdot \;m\left(v_{1B},\frac{v_{gB}}{l_{g}}\right)$ are the nonlinear dependent voltage sources on the magnetic ports.
7. $R_{feG}=R_{feR}=R_{feB}=l_{fe}/\left(\mu_{r,fe}\mu_{0}S_{fe}\right)$, are the reluctance of the steel magnetic circuit for each rod and for the symmetry of the object have been considered equals. It can be achieved from the magnetic circuit length, section and from the magnetic characteristics of the steel. In particular, $l_{fe}=49$ mm, $\mu_{r,fe}=500$ and $S_{fe}=94.2$ mm^2^.
8. $R_{leak}=1.216\times 10^{6}$ H^−1^ represents the leakage of magnetic flux between the top e bottom discs. Its value have been obtained with a minimization of the error between measured and simulated output voltages in case of optimal magnetic bias and one specific electric load inserted.
9. $R_{air}=l_{air}/\left(\mu_{0}S_{air}\right)$ is the reluctance of air-gap between the top steel disc and the permanent magnet. In particular, $l_{air}=1$ mm while the section has been considered equal to PM disc section, $S_{air}=S_{m}$.
10. $R_{m}=l_{m}/\left(\mu_{0}S_{m}\right)$ is the reluctance of the permanent magnet. It depends only by directly measurable quantities.
11. $l_{m}=20$ mm is the discs permanent magnet length, then directly measurable.
12. $S_{m}$ is the discs permanent magnet section, then directly measurable, in particular it have been used 10, 15 and 20 mm diameter of PM.
13. $M_{m}$ is the magnetization of neodymium PM and it is obtained from material datasheed (in this work it is considered equal to 1000 kA/m).
14. $R_{gG}=R_{gR}=R_{gB}=l_{g}/\left(\mu_{0}S_{g}\right)$, are the linear contribution of the total reluctance of the rods and have been considered equals.
15. $N_{2}=2000$ is the turn number of pickup coils, which is the same for each coil.
16. $v_{outG}=-\frac{d}{dt}N_{2}i_{gG}$, $v_{outR}=-\frac{d}{dt}N_{2}i_{gR}$ and $v_{outB}=-\frac{d}{dt}N_{2}i_{gB}$, are the nonlinear dependent voltage sources on the electrical ports.
17. $R_{coilG}=R_{coilR}=R_{coilB}=175\;\Omega$ is the pickup coil resistance are directly measurable and equals because same coils have been used.
18. $R_{load}$ is the applied electrical load.
